# *Neoehrlichia mikurensis*—A New Emerging Tick-Borne Pathogen in North-Eastern Poland?

**DOI:** 10.3390/pathogens12020307

**Published:** 2023-02-12

**Authors:** Magdalena Szczotko, Katarzyna Kubiak, Mirosław Mariusz Michalski, Leonardo Moerbeck, Sandra Antunes, Ana Domingos, Małgorzata Dmitryjuk

**Affiliations:** 1 Students’ Parasitology “Vermis” Science Club, Department of Medical Biology, Collegium Medicum, School of Public Health, University of Warmia and Mazury, 10-719 Olsztyn, Poland; 2 Department of Biochemistry, Faculty of Biology and Biotechnology, University of Warmia and Mazury in Olsztyn, Oczapowskiego 1A, 10-719 Olsztyn, Poland; 3 Department of Medical Biology, Collegium Medicum, School of Public Health, University of Warmia and Mazury in Olsztyn, Zolnierska 14c, 10-561 Olsztyn, Poland; 4 Department of Parasitology and Invasive Diseases, Faculty of Veterinary Medicine, University of Warmia and Mazury in Olsztyn, Oczapowskiego 13, 10-719 Olsztyn, Poland; 5 Global Health and Tropical Medicine, Institute of Hygiene and Tropical Medicine, NOVA University, 1349-008 Lisbon, Portugal; 6 Institute of Hygiene and Tropical Medicine, NOVA University, 1349-008 Lisbon, Portugal

**Keywords:** *Neoehrlichia mikurensis*, *Ixodes ricinus*, *Dermacentor reticulatus*, ticks, tick-borne pathogen, One Health

## Abstract

*Neoehrlichia mikurensis* is a new emerging tick-borne Gram-negative bacterium, belonging to the family Anaplasmataceae, the main vector of which in Europe is the tick *Ixodes ricinus*. *N. mikurensis* is responsible for neoehrlichiosis, occurring mostly in patients with underlying diseases. In the present study, a total of 348 *I. ricinus* and *Dermacentor reticulatus* ticks collected in north-eastern Poland were analyzed for the prevalence of *N. mikurensis.* A total of 140 questing ticks (124 of *I. ricinus* ticks and 16 *D. reticulatus*) collected with the flagging method and 208 ticks (105 and 103 *I. ricinus* and *D. reticulatus*, respectively) removed from dogs were selected for the study. cDNA (questing ticks) and total DNA (questing and feeding ticks) were analyzed by qPCR targeting the 16S rRNA gene of *N. mikurensis.* Positive samples were further analyzed by nested PCR and sequencing. The prevalence differed between ticks collected from vegetation (19.3%; 27/140) and ticks removed from dogs (6.7%; 14/208). The presence of the pathogen in questing and feeding *D. reticulatus* ticks was proven in Poland for the first time. In summary, our research showed that infections of ticks of both the most common tick species *I. ricinus* and *D. reticulatus* in north-eastern Poland are present and ticks collected from urban areas were more often infected than ticks from suburban and natural areas. The detection of *N. mikurensis* in *I. ricinus* and *D. reticulatus* ticks from north-eastern Poland indicates potential transmission risk for tick-bitten humans at this latitude.

## 1. Introduction

*Neoehrlichia mikurensis*, an emerging tick-borne intracellular pathogen, is an etiological agent of neoehrlichiosis, a severe systematic inflammatory syndrome [1]. The bacterium was classified as a member of the Anaplasmataceae family and named as *Candidatus* Neoehrlichia mikurensis in 2004 [2]. Recently, in 2019, the isolation of the bacterium in pure culture has been reported and its name has lost the prefix “*Candidatus*” [3]. In Europe, *Ixodes ricinus* is recognized as the main vector of this pathogen. Rodents are the reservoirs, and bacteria are widely distributed in wild and domestic mammals and birds. Prevalence of *N. mikurensis* in questing *I. ricinus* ticks collected in 18 European countries ranges from 0.1% (Denmark) to 24.3% (Hungary) [4]. Detection of *N. mikurensis* in female *I. ricinus* salivary glands suggests that this bacterium uses the “saliva transmission pathway” [5]. There is no proven evidence for the epidemiological relevance of *Dermacentor reticulatus* ticks in the transmission of the *N. mikurensis* [6,7]. However, two studies from Germany have shown that 7.7% of *D. reticulatus* ticks collected from small mammals and 0.08% questing ticks were *N. mikurensis* positive [7,8]. The first description of human neoehrlichiosis was published in 2010. Initially, it was assumed that this disease mainly affected people with immune deficiencies, but it has been confirmed to affect immunocompetent patients as well [9,10,11]. One of the important target cells of the infection is the vascular endothelium, and neoehrlichiosis patients with compromised B cell immunity present more severe inflammation than immunocompetent patients [12]. The symptoms of the disease are nonspecific, including joint pain, long-lasting fever, migrating pain, chills, nightly sweats, and risk of thromboembolic events are characteristic during *N. mikurenis* infection. Thrombosis afflicts not only superficial and deep veins, but it can also affect the arteries as well, causing arteritis or arterial embolism [11,13].

The aim of the present research was to detect *N. mikurensis* in questing and feeding *I. ricinus* and *D. reticulatus* ticks collected in the Olsztyn districts and its suburban areas. In line with the idea of “One Health”, results of this study will contribute to show the importance of ticks in the spreading of pathogens representing a threat to human health in north-eastern Poland.

## 2. Materials and Methods

### 2.1. Tick Collection

Ticks were collected from May to September of 2021 in urban and suburban areas of Olsztyn, the capital of the Warmia and Mazury region, in north-eastern Poland. Questing ticks collected from Purda (98 ticks), Zazdrość (18 ticks), Pieczewo (24 ticks), and feeding ticks collected from Jaroty (48 ticks removed from 26 dogs—average 1.84 ticks per dog) were used for further analyses. Ticks collected from two other locations, Dajtki and Zatorze, were randomly chosen and divided into four groups of 20 individuals. In both locations, 80 ticks were randomly selected from 253 (1.91 ticks per dog) and 339 ticks (2.04 ticks per dog) from Dajtki and Zatorze, respectively.

Questing ticks in Purda (suburban recreational area by the lake, natural biotope), Zazdrość (suburban mid-forest parking, natural biotope), and in Pieczewo (district of Olsztyn city, municipal dog walking area) were collected during the daytime between 9 a.m. and 4 p.m. by one person for at least 30 min using the standard flagging method and preserved in stayRNA^TM^ buffer (A&A Biotechnology, Gdynia, Poland). Feeding ticks, attached to owned dogs‘ skin and found on dog fur were removed in veterinary clinics located in three Olsztyn districts: Zatorze (a housing estate near the city forest, highly urbanized area), Dajtki (green areas of single-family houses, less urbanized area), Jaroty (the largest housing estate, highly urbanized area) during follow-up visits and placed in 70% ethanol for further molecular investigation. The collected ticks were identified by species, sex, and developmental stage using a taxonomic key [14].

### 2.2. Extraction of Total Nucleic Acids

**Figure 1:**
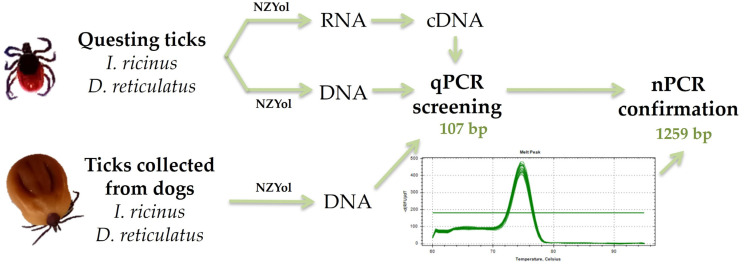
Scheme of the experimental methodology. Questing ticks or ticks retrieved from dogs were used to detect the presence of *Neoehrlichia mikurensis* using qPCR and nPCR methods.

The ticks’ RNA/DNA was extracted from the whole individuals using the NZYol reagent (NZYTech, Lisbon, Portugal) according to the manufacturer’s instruction (Figure 1). The amount of obtained RNA was determined by Qubit Fluorometric Quantitation (Thermo Fisher Scientific, Waltham, MA, USA). To evaluate the quality of the obtained DNA and to evaluate PCR inhibition, 460 bp fragments of the tick 16S rDNA gene were amplified by conventional PCR in 25 μL reactions using the Supreme NZYTech Taq 2× Green Master Mix (NZYTech, Lisbon, Portugal). Primer sequences and conditions previously described by Black and Piesman [15] (Table 1) using a T100 BioRad Thermal Cycler (BioRad, Hercules, CA, USA) were used.

### 2.3. cDNA Synthesis and qPCR Detection of Neoehrlichia mikurensis in Questing Ticks

Total RNA extracted from the questing ticks were used for cDNA synthesis using the iScript cDNA Synthesis Kit (Bio-Rad, Hercules, CA, USA), following the manufacturer’s protocol. cDNA and DNA were screened for the presence of *N. mikurensis* using qPCR (quantitative PCR) (Figure 1). To amplify a 107-bp-long 16S rRNA gene amplicon, reactions were prepared in triplicates in 96-well plates, using the primers NEO_16S_F/NEO_16S_R (Table 1) [16]. The reactions consisted of 5 μL of SYBR Green mix (Bio-Rad, Hercules, CA, United States), 0.2 μL of each primer (10 μM), 1 or 3 μL of template (cDNA or DNA, respectively) and topped up with RNAse-free water to 10 μL. The reactions were run on a CFX Connect Real-Time PCR Detection System (BioRad) equipped with FAM and HEX filter sets and further analyzed with CFX Manager software (BioRad) or on a QuantStudio™ 3 Real-Time PCR System of Applied Biosystems (Thermo Fisher Scientific). The cycling conditions consisted of an activation step at 95 °C for 3 min, followed by 45 cycles of 95 °C for 15 s, 60 °C for 30 s, and 72 °C for 30 s. Immediately after, melting curve analyses were performed by cooling to 60 °C for 1 min, and subsequent heating to 95 °C at 0.5 °C/min with continuous fluorescence recording. Eight-fold serial dilutions were used to construct standard curves with synthetized gBlocks^®^ Gene Fragment (IDT-Integrated DNA Technologies, Leuven, Belgium) encompassing a 106 bp of the *N. mikurensis* 16S ribosomal gene. Cycle threshold (Ct) values below 40 and melting temperatures between 74.5 and 75.5 °C (BioRad System), or 79.7 and 80.7 °C (Applied Biosystems) were considered as *N. mikurensis*-positive. Triplicates with Cq (quantification cycle) differences greater than 0.5 were excluded from the analysis. The amplicon identity was confirmed by sequencing with the Sanger method at StabVida (Lisbon, Portugal).

### 2.4. qPCR Detection of Neoehrlichia mikurensis in Ticks Removed from Dogs

Genomic DNA extracted from ticks retrieved from dogs was screened with qPCR to detect the pathogen (Figure 1). DNA was analyzed in triplicate as described above. Three μL of DNA template was used.

### 2.5. Nested PCR Assay

Positive samples for *N. mikurensis* in the qPCR assay were further validated with a nested PCR assay (Figure 1). The nPCR included primers targeting the 16S rRNA gene of *N. mikurensis,* to amplify a 1259-bp-long amplicon (Table 1). The 25 μL reactions of the first round of amplification consisted of 12.5 μL Supreme NZYTech Taq 2× Green Master Mix (NZYTech), 0.5 μL of each of the primers Neo_16S_95_F/Neo_16S_1393_R (10 μM, Table 1), and 9 μL RNAse free water.

### 2.6. DNA Sequencing and Data Analysis

A randomly selected representative number of nested PCR products positive for *N. mikurensis* (*n* = 4: *I. ricinus* and *D. reticulatus*, questing and feeding), were purified using the quick DNA clean-up NZYGelpure kit (NZYTech) according to the manufacturer’s protocol and bidirectionally sequenced with the Sanger method at StabVida (Lisbon, Portugal). The obtained nucleotide sequences were edited in BioEdit software [17] and compared with data registered in the GenBank database (https://www.ncbi.nlm.nih.gov/genbank/index.html, accessed on 26 August 2022) using the BLAST-NCBI program (http://www.ncbi.nlm.nih.gov/BLAST/, accessed on 20 October 2022). Consensus sequences of the fragment of *N. mikurensis* 16 S rRNA gene were deposited in the GenBank database and registered under the accession numbers OP269534-OP269537. Representative sequences obtained in this study and the most similar sequences chosen from GenBank were used in phylogenetic analysis. The phylogram was constructed based on the neighbor-joining method and the Maximum Composite Likelihood as a distance method. The topology of the phylogram was evaluated using the bootstrap method with 1000 replicates. Phylogenetic analysis was conducted using MEGA X software (Penn. State University, Philadelphia, PA, USA).

### 2.7. Statistics

A statistical analysis was performed using a two-sided Fisher’s exact test (Prism 6 program, GraphPad Software, San Diego, CA, USA). The prevalence of pathogens was calculated with 95% confidence intervals (95% CI) using the “exact” interval by Clopper and Pearson. A Chi-square test was used to check whether there was a relationship between groups of tested tick (χ^2^). Values of *p* < 0.05 were considered statistically significant.

## 3. Results

### 3.1. Tick Collection

In total, 348 tick individuals were subjected to molecular analyses. The questing ticks constituted 40.2% of them (*n* = 140). *I. ricinus* ticks (124 individuals: 81 nymphs, 18 females, and 25 males) dominated over *D. reticulatus* (16 individuals: 9 females and 7 males). *I. ricinus* were collected in all three surveyed sites, and *D. reticulatus* was collected only in the Pieczewo district (Table 2). From the 208 adult ticks removed from dogs and analyzed, 105 were morphologically identified as *I. ricinus* (60 engorged or semi-engorged females and 45 males) and 103 were identified as *D. reticulatus* (58 engorged or semi-engorged females and 45 males). No nymphs were found among the ticks studied in this group (Table 2).

### 3.2. Molecular Identification of Neoehrlichia mikurensis in Ticks

Overall, 19.3% of questing ticks were found to be infected with *N. mikurensis* this study (Table 2). Among questing ticks, statistically significant differences were noted for both tested species of ticks (χ^2^ = 15.9; *p* = 0.00007). Among the less numerous, *D. reticulatus,* as many as 56.3% were infected, while in *I. ricinus* ticks the infection was at the level of 14.5% (Table 2). In the group of questing ticks, infected ticks were recorded only in the urban area of Pieczewo, the district of Olsztyn, and in the suburban recreational area of Purda with a natural biotope (Table 2). Among the eight adult ticks of *I. ricinus* caught around the Pieczewo district, molecular detection of *N. mikurensis* was noted in four (50.0%; 95% CI: 15.7–84.3). In Purda, where 98 nymph and adult *I. ricinus* ticks were caught, the percentage of infected ticks was lower, reaching 14.3% (14/98; 95% CI: 8.0–22.8). The differences demonstrated for the ticks obtained from Purda and Pieczewo were statistically significant (χ^2^ = 6.7, *p* = 0.01). *D. reticulatus* ticks were not collected in Purda and Zazdrość (Table 2).

Amplification of *N. mikurensis* DNA in ticks removed from owned dogs was 6.7% (Table 3). In this group of ticks, only feeding females were found to be positive (13.3% and 10.3% for *I. ricinus* and *D. reticulatus*, respectively; Table 3). The differences found for both feeding female ticks were not statistically significant for *p* < 0.05 (χ^2^ = 0.3, *p* = 0.62). Generally, the rate of positives for both species of feeding ticks was 7.6% for *I. ricinus* and 5.8% for *D. reticulatus* (χ^2^ = 0.27, *p* = 0.61). Positive samples were not obtained from ticks of dogs in the Dajtki district (Table 3).

In the other two highly urbanized districts of Olsztyn, the infection was at a similar level. In Zatorze, 7.5% (3/40; 95% CI: 1.6–20.4) of the screened *I. ricinus* and 10% (4/40; 95% CI: 2.8–23.7) of the *D. reticulatus* ticks were found to be infected. In Jaroty, 20.0% (5/25; 95% CI: 6.8–40.7) of *I. ricinus* and 8.7% (2/23; 95% CI: 1.1–28.0) of *D. reticulatus* ticks were infected. There were no statistically significant differences (χ^2^ = 2.23, *p* = 0.14 and χ^2^ = 0.03, *p* = 0.87 for *I.ricinus* and *D. reticulatus* ticks, respectively). The analysis demonstrated a higher pathogen infection in questing ticks (19.3%; 27/140, Table 2) than in arachnids removed from hosts (6.7%; 14/208, Table 3). The differences noted for both groups were statistically significant for *p* < 0.05 (χ^2^ = 12.7, *p* = 0.0004).

### 3.3. Molecular Relationships between Neoehrlichia mikurensis Identified in the Study and Accessions from GenBank

**Figure 2:**
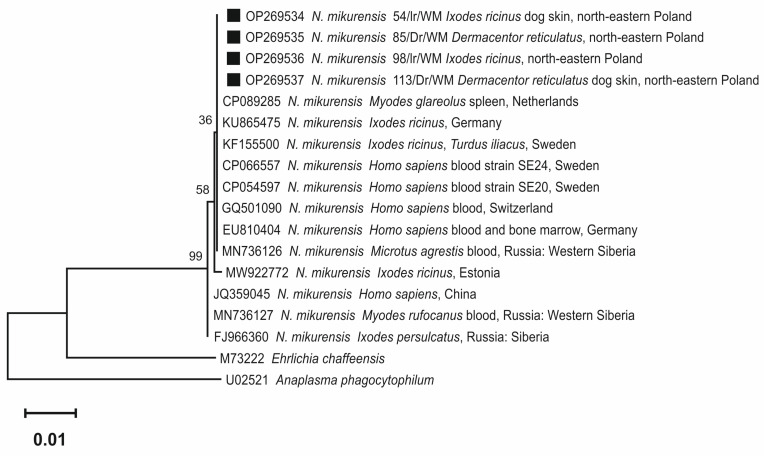
Molecular relationships based on the sequences of the 16S rRNA gene of *Neoehrlichia mikurensis* (751 bp after alignment) identified in the study. The phylogenetic tree was constructed using the maximum likelihood method and the Kimura 2-parameter model as a distance method. Numbers at the tree nodes indicate percent of bootstrap value from 1000 replicates. The tree is drawn to scale, with branch lengths measured in the number of base substitutions per site. The analyses and phylogram construction were conducted in MEGA X software [18]. The sequences obtained in this study were labelled with black symbols. *Ehrlichia chaffeensis* and *Anaplasma phagocytophilum* were used as outgroups.

Molecular analysis of the 751 bp fragment of the 16S rRNA gene of *N. mikurensis* obtained from *I. ricinus* and *D. reticulatus* questing and feeding on hosts showed that all sequences were identical (Figure 2).

Analyzed sequences had 100% nucleotide similarity to sequences of *N. mikurensis* isolated from the blood of patients from Sweden (GenBank: CP066557 and CP054597), Switzerland (GenBank: GQ501090) and Germany (GenBank: EU810404).

Similar sequences were also detected in questing *I. ricinus* ticks in Germany (GenBank: KU8654750 and from *I. ricinus* feeding on birds in Sweden (GenBank: KF155500). Our isolates clustered also with *N. mikurensis* sequences obtained from biological material from wild *Microtus* rodents in the Netherlands (GenBank: HM045824) and in Western Siberia in Russia (GenBank: MN736126) (Figure 2).

## 4. Discussion

Ticks transmit numerous pathogens threating human and animal health, and therefore have a significant impact on public health. Environmental factors and animal hosts are important factors determining the prevalence/epidemiology of tick-borne diseases (TBD). The “One Health” approach is especially relevant for the effective control and prevention of zoonoses (diseases that can spread between animals and humans), such as some TBDs. Thus, changes in tick and TBD transmission affecting livestock, companion animals, and humans should be particularly monitored [19].

The growing global awareness allied with advances in molecular methods have contributed to the discovery of more new pathogenic microorganisms, such as the new-emerging pathogen, *N. mikurensis*. According to the results of these studies, both the most common species of tick, *I. ricinus,* and the second most frequent tick species in Poland, *D. reticulatus,* [20], were infected. The detection of *N. mikurensis* in *D. reticulatus* ticks has not been demonstrated so far in Poland [21,22]. It is known that *D. reticulatus* adults bite humans less frequently than *I. ricinus*. However, its ability to transmit several pathogenic microorganisms has been proven [23]. Although there is no evidence that *D. reticulatus* ticks have epidemiological relevance in the transmission of *N. mikurensis* [6,7], based on our observations and those published by German researchers [7,8] it can be suggested that *D. reticulatus* ticks participate in the circulation of the pathogen in the environment. However, more studies are necessary to investigate the potential role of this tick species in the bacteria’s life cycle, as it cannot be ruled out that *D. reticulatus* ticks acquire the pathogen while feeding without passing it on to next hosts.

On the other hand, significant differences were noted between the tick collection areas. *N. mikurensis* was not found in two tick harvesting habitats, Zazdrość (questing ticks) or Dajtki (ticks removed from dogs). Both areas are poorly urbanized. Hence, it can be suggested that the pathogen spreads faster in north-eastern Poland in urbanized areas with a greater concentration of people and domestic animals. Additionally, Kowalec et al. [20] observed a higher prevalence of *N. mikurensis* in *I. ricinus* ticks collected from urban areas than from natural biotopes.

It is important to denote that in the present study, none of the tested *I. ricinus* and *D. reticulatus* males collected from dogs were infected with *N. mikurensis*. This is surprising, since the questing males analyzed were infected. Similarly, in the research by Król et al. [24], only engorged female *I. ricinus* ticks isolated from dogs were infected. Further investigation needs to be conducted to clarify this observation, using more male ticks from different regions.

As previously mentioned, our analysis showed a higher pathogen presence in questing ticks (19.3%) than in those detached from dogs (6.7%). This may be due to the underestimation of the samples. For questing ticks, cDNA was screened using the qPCR method which appears to be more sensitive than the DNA/qPCR screening used for ticks removed from pets. These results are similar to other studies performed in Europe, where 4.1–8.1% of ticks isolated from dogs and 0.1–24.3% ticks from vegetation were positive for *N. mikurensis* [4,9,21,24,25].

Analysis of the fragment of the 16S rDNA of *N. mikurensis* obtained from ticks from north-eastern Poland showed their high homology with other isolates, mainly from Europe. This confirms low heterogeneity in the 16S rRNA gene of this pathogen in European populations of ticks revealed in other studies [21,26,27]. The identified genetical variant of *N. mikurensis* was identical to the strains causing human infection in patients from Sweden [28], Switzerland [29], and Germany [30]. Multilocus sequence assay [MLSA] among 12 European human isolates indicated three genotypes of *N. mikurensis* [26]. However, a low genetic diversity in the analyzed loci was shown, indicating that the strains infecting humans in Europe were quite similar. The first description of neoehrlichiosis in humans was published only in 2010 [10]. In the following years, several cases of this disease were described in Europe: in Switzerland, Germany, the Czech Republic, and Sweden. In most cases, infection was found in humans showing a weakened immune system [26,29,30,31,32,33,34,35,36]. In turn, the detection of the bacteria in donated blood in south-eastern Sweden indicates the need to raise clinical awareness of this issue. Although *N. mikurensis* was detected in 0.7% of the donated blood, no transfusion-associated infection was detected, even though several recipients were at high risk of severe neoehrlichiosis. These results, however, justify further research and continuous monitoring of donated blood since apparently asymptomatic cases may occur [16]. In Poland, there are no registered cases of disease caused by *N. mikurensis* bacteria, so far. Throughout the country, only asymptomatic infections have been found in healthy individuals in molecular screening studies [9,37]. However, considering the relatively high prevalence of the pathogen in ticks found in this study and the dynamics of changes in the epidemiology of TBD, such a risk cannot be ruled out. In view of the above, it seems reasonable to conduct detailed studies to determine the percentage of infections in vectors and reservoirs to determine the geographical distribution of this pathogen. The results of this study indicate that the two most common tick species in Poland show the presence of the emerging pathogen *N. mikurensis*. It may be considered as quite disturbing for humans at risk of tick attack in this region of Europe. Results obtained in this study indicate that ticks carrying *N. mikurensis* may be found within the urban premises of the study area, which is an important factor regarding public health risk for TBD. In the future, the collected data will allow us to mark the endemic places of *N. mikurensis* and determine the potential threat to humans.

## 5. Conclusions

The presented screening studies showed that infection with the bacterium *N. mikurensis* concerns both the most common tick species *I. ricinus* and *D. reticulatus* in north-eastern Poland. Although *I. ricinus* is the main vector of the pathogen in Europe, for the first time in Poland we demonstrated the detection of *N. mikurensis* also in questing and feeding *D. reticulatus*. On the other hand, ticks of both species collected from urban areas were more often infected than ticks from suburban and natural areas. This may suggest a significant role of companion animals in the spread of the pathogen representing a real threat of neoehrlichiosis for people living in agglomerations.

## Figures and Tables

**Table 1:** Primers used in the study and expected fragment length.

Method	Target Gene	Primer Name	Primer Sequence 5′-3′	Product Size [bp]	Reference
PCR	16S rDNA	Tick_16S_F	CTGCTCAATGATTTTTTAAATTGCTGTGG	460	[15]
		Tick_16S_R	CCGGTCTGAACTCAGATCAAGT		
qPCR	16S rRNA	Neo_16S_F	GTAAAGGGCATGTAGGCGGTTTAA	107	[16]
		Neo_16S_R	TCCACTATCCTCTCTCGATCTCTAGTTTAA		
nPCR	16S rRNA	Neo_16S_95_F	TTAGTGGCAGACGGGTGAGTAATG	1321	
		Neo_16S_1393_R	TCCTTACGGTTAGCTCACCAGCTT		
		Neo_16S_127_F	TCTGCCTAGTAGTATGGAATAGCTG	1259	
		Neo_16S_1363_R	AAACCAATTTCCAGGGCATGACGG		

**Table 2:** Prevalence of *Neoehrlichia mikurensis* in questing and feeding *Ixodes ricinus* and *Dermacentor reticulatus* ticks collected in north-eastern Poland.

Collection Site	*Ixodes ricinus*			*Dermacentor reticulatus*	
	Nymph; % (95% CI)	Female; % (95% CI)	Male; % (95% CI)	Female; % (95% CI)	Male; % (95% CI)
**Purda**	9/70; 12.8 (6.1–23.0)	1/11; 9.1 (0.2–41.3)	4/17; 23.5 (6.8–49.9)	–	–
**Zazdrość**	0/11; 0.0 (0.0–28.5)	0/3; 0.0 (0.0–70.8)	0/4; 0.0 (0.0–60.2)	–	–
**Pieczewo**	–	3/4; 75.0 (19.4–99.4)	1/4; 25.0 (0.6–80.6)	3/9; 33.3 (7.5–70.1)	6/7; 85.7 (42.1–99.6)
**Subtotal**	9/81; 11.1 (5.2–20.0)	4/18; 22.2 (6.4–47.6)	5/25; 20.0 (6.8–40.7)	3/9; 33.3 (7.5–70.1)	6/7; 85.7 (42.1–99.6)
	18/124 (14.5%; 95% CI: 8.8–22.0)			9/16 (56.3%; 95% CI: 29.9–80.2)	
**Total**	**27/140 (19.3%; 95% CI: 13.1** **–** **26.8)**				

**Table 3:** Prevalence of *Neoehrlichia mikurensis* in feeding *Ixodes ricinus* and *Dermacentor reticulatus* ticks collected in north-eastern Poland.

Collection Site	*Ixodes ricinus*		*Dermacentor reticulatus*	
	Female; % (95% CI)	Male; % (95% CI)	Female; % (95% CI)	Male; % (95% CI)
**Dajtki**	0/20; 0.0 (0.0–16.8)	0/20; 0.0 (0.0–16.8)	0/20; 0.0 (0.0–16.8)	0/20; 0.0 (0.0–16.8)
**Zatorze**	3/20; 15.0 (3.2–37.9)	0/20; 0.0 (0.0–16.8)	4/20; 20.0 (5.7–43.7)	0/20; 0.0 (0.0–16.8)
**Jaroty**	5/20; 25.0 (8.7–49.1)	0/5; 0.0 (0.0–52.2)	2/18; 11.1 (1.4–34.7)	0/5; 0.0 (0.0–52.2)
**Subtotal**	8/60; 13.3 (5.9–24.6)	0/45; 0.0 (0.0–7.9)	6/58; 10.3 (3.9–21.2)	0/45; 0.0 (0.0–7.9)
	8/105 (7.6%; 95% CI: 3.3–14.5)		6/103 (5.8%; 95% CI: 2.2–12.2)	
**Total**	**14/208 (6.7%; 95% CI: 3.7** **–** **11.0)**			

## Data Availability

The data presented in this study are contained within the article.
